# Performance Evaluation of Deep Learning Image Classification Modules in the MUN-ABSAI Ice Risk Management Architecture

**DOI:** 10.3390/s25020326

**Published:** 2025-01-08

**Authors:** Ravindu G. Thalagala, Oscar De Silva, Dan Oldford, David Molyneux

**Affiliations:** 1 Faculty of Engineering and Applied Science, Memorial University of Newfoundland (MUN), St. John’s, NL A1B 3X5, Canada; oscar.desilva@mun.ca (O.D.S.); david.molyneux@mun.ca (D.M.); 2 American Bureau of Shipping (ABS), St. John’s, NL A1B 3X5, Canada; dandford@eagle.org

**Keywords:** sea ice risk mitigation, ice classification, sea ice images, deep learning, YOLOv8

## Abstract

The retreat of Arctic sea ice has opened new maritime routes, offering faster shipping opportunities; however, these routes present significant navigational challenges due to the harsh ice conditions. To address these challenges, this paper proposes a deep learning-based Arctic ice risk management architecture with multiple modules, including ice classification, risk assessment, ice floe tracking, and ice load calculations. A comprehensive dataset of 15,000 ice images was created using public sources and contributions from the Canadian Coast Guard, and it was used to support the development and evaluation of the system. The performance of the YOLOv8n-cls model was assessed for the ice classification modules due to its fast inference speed, making it suitable for resource-constrained onboard systems. The training and evaluation were conducted across multiple platforms, including Roboflow, Google Colab, and Compute Canada, allowing for a detailed comparison of their capabilities in image preprocessing, model training, and real-time inference generation. The results demonstrate that Image Classification Module I achieved a validation accuracy of 99.4%, while Module II attained 98.6%. Inference times were found to be less than 1 s in Colab and under 3 s on a stand-alone system, confirming the architecture’s efficiency in real-time ice condition monitoring.

## 1. Introduction

The ongoing retreat of Arctic sea ice, driven by climate change, has led to the opening of new maritime routes through the polar regions, presenting both opportunities and challenges [1]. As Arctic shipping routes expand faster than earlier climate models predicted, they offer significant economic benefits by reducing travel distances between major global ports and providing access to previously untapped natural resources [2]. However, these benefits are accompanied by the challenges posed by the harsh and dynamic ice conditions in the Arctic. The presence of various forms of sea ice, including thick multi-year ice, ice floes, and icebergs, poses a threat to the safety of vessels navigating these waters [3,4]. As such, the need for effective and reliable navigation systems in these environments has become critical.

Mitigating the risks associated with ice navigation is essential for ensuring the safety of vessels and their crews. Traditionally, ice risk quantification has relied on well-established synthetic aperture radar (SAR) image-based strategies [5] and onboard RADAR systems [6,7]. Techniques such as the watershed algorithm [8], curvelet transform [9], and gradient vector flow (GVF) snake algorithm [10] have been employed to extract ice floe information from SAR data [11]. However, interference clutter in SAR imagery often limits the reliability of these methods. Recent advancements, such as the dualistic cascade convolutional neural network (DCCNN), have improved detection accuracy for SAR-based ship detection by utilizing polarization characteristics and enhanced feature extraction [12]. Such developments indicate the potential for similar advancements in SAR-based ice detection.

In contrast to SAR and RADAR systems, onboard cameras provide a cost-effective solution for the continuous observation of sea ice. These in situ digital images, compared to large-scale satellite data, capture localized and high-resolution details of surrounding ice conditions [13]. The specific and detailed information derived from these images, including ice concentration, size, and temporal trends, can enhance situational awareness and facilitate proactive measures to mitigate navigational risks [14]. The growing availability of diverse datasets for ship detection and classification tasks [15] highlights the need for developing similar benchmark datasets dedicated to ice navigation. These datasets would support the application of advanced deep learning techniques, improving the accuracy and efficiency of ice risk quantification methods.

Understanding the scene from onboard camera images has significantly developed with Artificial Intelligence (AI) [7,14,16,17]. Traditional AI systems that utilize machine learning techniques such as support vector machines (SVM) [18], fuzzy k-means clustering [19], and object-based random forest (ORF) methods [20] face several limitations. These approaches rely heavily on manual feature engineering, which is time-consuming and requires domain expertise, often leading to limited generalization and adaptability. Additionally, they struggle with complex, high-dimensional data and can be sensitive to noise, resulting in reduced model performance in real-world applications [21]. Furthermore, the scalability and flexibility of these traditional methods are often inadequate for handling large, dynamic datasets, requiring frequent retraining and tuning [22].

**Figure 1:**
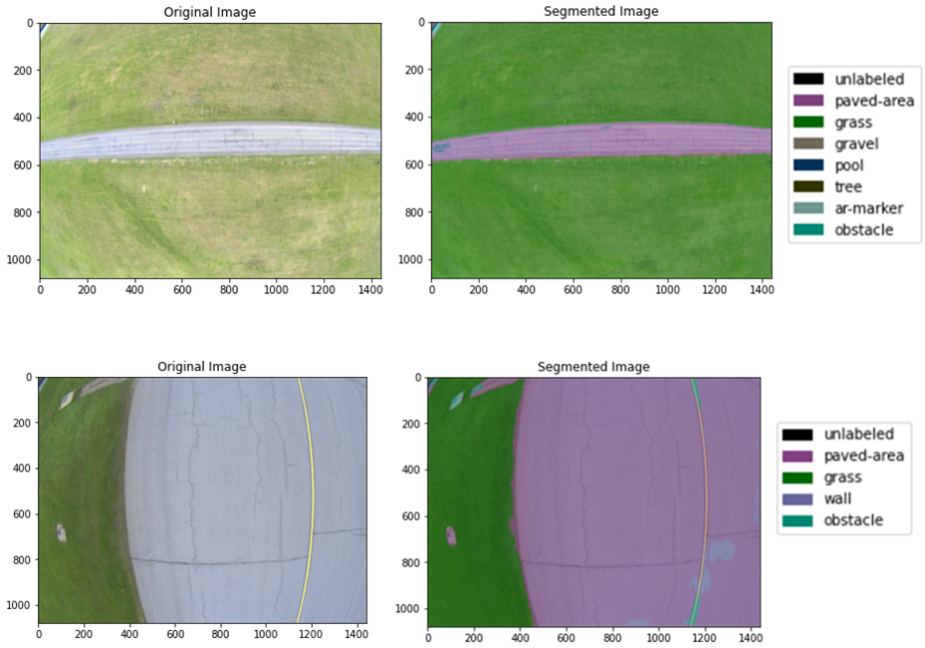
Object detection and classification typically used in autonomous full-scale aerial applications carried out using the datasets from our previous work [36].

Recent advancements in AI have increasingly focused on deep learning (DL) as an alternative to traditional machine learning techniques [23,24]. DL represents a subset of machine learning, characterized by its ability to automatically learn and extract significant features from raw data. Unlike traditional methods that rely on manual feature engineering, DL models operate autonomously, identifying patterns and details within the data without the need for explicit guidance. This capacity for understanding and capturing key information makes DL particularly well-suited for addressing complex tasks. Convolutional neural networks (CNNs) [25,26,27], a widely used DL architecture, have achieved considerable success in various applications, including semantic image segmentation [13], object detection [26], and tracking [28], across diverse domains. DL was shown to be capable of addressing the challenges posed by nonlinear data relationships in maritime applications, such as wave spectra retrieval [29]. For instance, the SAR2WV model demonstrates how deep learning can improve the quality of wave spectra retrieval by mapping nonlinear relationships. DL models increase accuracy and enable comprehensive scene understanding by extracting high-level semantic information. DL techniques have seen heavy application in autonomous driving systems (see Figure 1 [30,31] as well as in aerial platforms such as drones [32,33,34]. Current autonomous driving systems primarily utilize discriminative AI methods [35], a class of techniques focused on learning the boundaries between different classes in data. Discriminative models, such as those based on CNNs, operate accurately for tasks such as classification and regression by directly mapping inputs to labels. These models have been widely used in enabling the use of robust perception systems in autonomous vehicles, where the accurate identification of objects, pedestrians, and other vehicles is critical to safe navigation.

However, recent AI advancements have increasingly incorporated generative AI techniques with discriminative methods [37]. Generative AI involves models that learn to generate new data samples that resemble the training data. Unlike discriminative models, which focus on distinguishing between classes, generative models aim to understand the underlying data distribution [38]. This approach has opened new possibilities for AI, including creating realistic synthetic data, enhancing data augmentation, and enabling more sophisticated decision-making processes. Generative models, such as Generative Adversarial Networks (GANs) [39] and Variational Autoencoders (VAEs) [40], have shown promise in applications ranging from image synthesis to anomaly detection.

**Figure 2:**
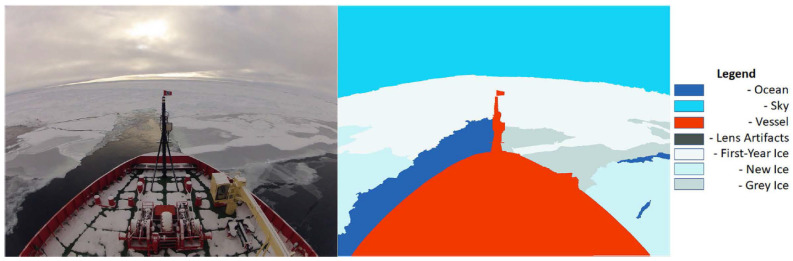
Sematic segmentation of in situ ice field using PSPNet101 in our previous work [13]. Reproduced with permission from [Benjamin Dowden], [Sea Ice Classification via Deep Neural Network Semantic Segmentation]; published by [IEEE], [2020].

In the ice navigation domain, discriminative supervised learning methods have been widely utilized, with notable approaches including DenseNet [6], Modified VGG-16 [41], U-Net with Dual-Attention Mechanism [42], and ICENET [43]. These studies primarily focus on synthetic aperture radar (SAR) sea ice images, which provide large-scale data but often lack the spatial specificity required for certain navigation tasks. In contrast, in situ digital sea-ice images captured by onboard cameras, as shown in Figure 2, offer high-resolution, localized insights into the surrounding ice conditions. Unlike satellite images, onboard cameras can capture fine-grained details, making them particularly useful for assessing specific ice formations and conditions [6,13].Despite the potential of in situ images, the development of robust deep learning models for these data are hindered by the lack of labeled datasets. Established applications of deep learning, such as autonomous driving, pedestrian tracking, and object recognition, have benefited significantly from the availability of large, well-labeled datasets. However, in the context of polar ice navigation, there are no open-source labeled datasets specifically designed for classifying ice types. This gap poses a significant challenge to advancing discriminative AI models for sea ice classification [21,44]. The importance of datasets is well-documented in related fields, such as ship detection and classification, where benchmark datasets have driven advancements in deep learning techniques [15]. The lessons from these fields underscore the necessity of creating large datasets for the challenges of ice navigation. Such datasets would enable the development of accurate and generalizable deep learning models, facilitating safer navigation in polar environments.

In the ice navigation domain, a discriminative AI model is essential for performing tasks critical to ice risk management. These tasks include determining the ship’s navigation direction based on camera positioning; preprocessing camera images to filter out unusable data (such as images affected by low light conditions, fog, or lens artifacts); and detecting ice regions, open-water areas, other vessels, and icebergs. Furthermore, the system must identify ice types and concentrations, as well as detect and track ice floes, to plan navigation routes that avoid hazardous ice.

Therefore, this paper proposes an Arctic ice risk management architecture that utilizes onboard camera feeds and integrates existing deep learning models and tools for its efficient implementation. The contributions of this work include the creation of a 15,000-image ice dataset sourced from public data and the Canadian Coast Guard; the development of modules for ice classification, risk assessment, ice floe tracking, and ice load calculations; and the evaluation of the YOLOv8n-cls model for fast and efficient ice classification. Additionally, this paper provides a comparative analysis of platforms, including Roboflow, Google Colab, and Compute Canada, to assess their suitability for tasks such as image preprocessing, model training, and real-time inference.

## 2. Ice Risk Management Architecture

**Figure 3:**
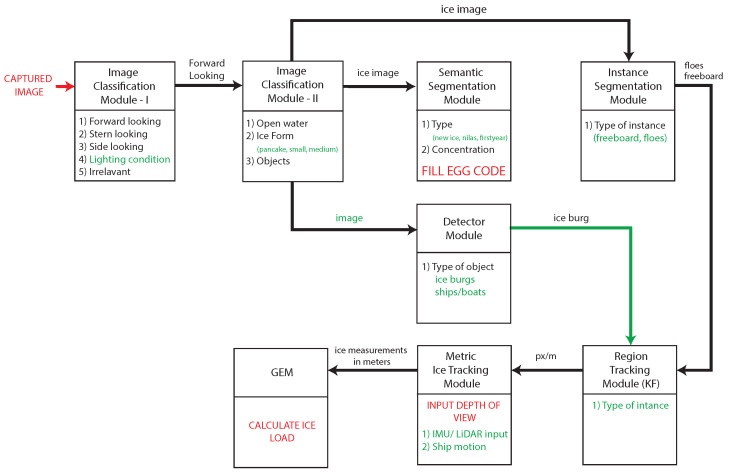
Details of the AI sub-modules within the architecture. The image feed is processed by the Ice Classification Module I, after which the images pass through each subsequent sub-module. The numbered lists within each box represent the specific classes or outputs generated by that module. A GPU-based event mechanics model (GEM) [45] is denoted as GEM.

The proposed ice detection and tracking architecture introduces modules designed to utilize onboard camera feeds for multiple ice navigation purposes. These include detecting various ice types and formations through semantic segmentation, tracking ice floes via instance segmentation, and calculating ice pressure using object detection and tracking techniques. A visual representation of the architecture, along with a summary of each module, is presented in Figure 3. Detailed descriptions of these modules are provided in the following sections.

### 2.1. Image Classification

The image classification modules serve as preprocessors for the subsequent semantic segmentation and instance segmentation modules. The image feed from the onboard cameras is first directed into these classification modules. This ensures that only the relevant images necessary for the later processing stages are passed through the system. By filtering out corrupted or unprocessable images, these modules protect the segmentation modules from potential overload and maintain the overall efficiency and reliability of the system.

#### 2.1.1. Image Classification Module-I (ICM-I)

**Figure 4:**
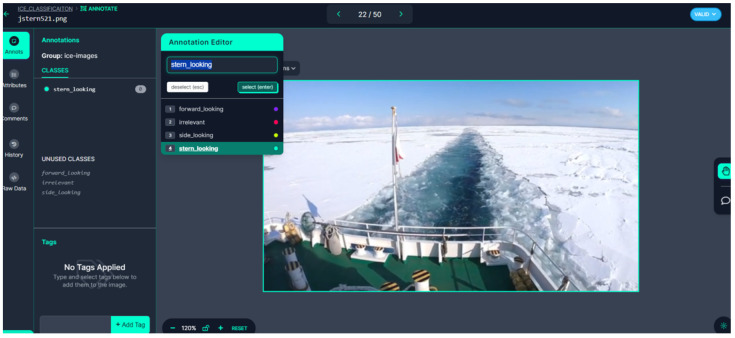
Graphical image annotation tool found in Roboflow [48].

This module processes the real-time image feed from the onboard cameras, evaluating each image for relevance by determining if it contains ice and assessing whether the image quality, particularly lighting conditions, is sufficient for accurate ice detection. The images are classified into five categories based on the scene as follows: forward-looking; side-looking; stern-looking, i.e., backward-facing direction from the ship deck, see Figure 4; lighting condition; and irrelevant. The light condition class identifies variations in illumination levels in the image, including low-light nighttime scenes, high-glare daytime images, and other situations where lighting impacts visibility. Meanwhile, the irrelevant class includes images that do not contribute to ice navigation, such as sky views, internal ship components, blurred or obscured scenes, and other content unrelated to the operational environment. As a preprocessing step, this module ensures that only images containing ice and those with adequate lighting are passed to the next stage for further analysis. The output of this module includes images that fall into the forward-looking category for immediate processing. Additionally, images classified as side-looking and stern-looking, if containing ice, are flagged for future ice load calculation modules.

#### 2.1.2. Image Classification Module-II (ICM-II)

This module processes forward-looking images, distinguishing between images of open water (i.e., no ice present); images containing ice; and images featuring distinct objects such as icebergs, ships, and boats. The module outputs three types of images as follows: those containing ice, those depicting open water, and those with distinct objects. Ice-containing images are subsequently routed to the semantic segmentation modules for ice type identification and to the instance segmentation modules for ice floe determination. Images with distinct objects are forwarded to the object tracking module for tracking purposes, while open-water images are filtered out from the system and discarded without further processing.

#### 2.1.3. Semantic Segmentation Module

Semantic segmentation is an advanced technique in machine vision that goes beyond simple object classification [13]. It not only identifies objects within an image but also labels each pixel with a class identifier specific to that object type. This method allows for detailed understanding and analysis of complex scenes, assigning distinct categories such as people, buildings, and vehicles, which are critical for applications like urban scene recognition. In the proposed system architecture, semantic segmentation is used to detect ice types and ice concentrations. The output of this module include ice type and ice concentration, which support the Risk Index Outcome (RIO) calculations using Polar Operational Limitations Assessment Risk Indexing System (POLARIS) guidelines. This module is implemented in the smartphone application that has been developed by the American Bureau of Shipping (ABS)—Harsh Environment Technology Center (HETC) [46].

#### 2.1.4. Instance Segmentation Module

Instance segmentation [47] is another advanced machine vision technique widely used in applications such as self-driving vehicles. Unlike semantic segmentation, which classifies each pixel under a broad category, instance segmentation identifies and categorizes each instance of multiple object classes independently. For instance, in self-driving systems, this allows the system to distinguish between individual vehicles on the road, not just recognizing them as vehicles, but also identifying each one separately, assigning a unique label to it. Similarly, in sea ice classification, instance segmentation can differentiate between various ice forms, marking them individually, even if they belong to the same class. For example, one image may contain multiple small floes, and instance segmentation can assign distinct labels to all the small floes that were detected. This module takes pack ice images as input and output individually segmented ice floes, enabling multiple ice floe tracking in the preceding modules.

#### 2.1.5. Object Detection Module

Object detection modules are commonly used in the autonomous navigation of vehicles and drones to avoid obstacles by detecting objects such as pedestrians, vehicles, trees, and buildings. In the proposed architecture, an object detection module is used to identify distinct objects such as ice burgs, ships, and boats that are present in the input pack ice image. Detected objects can be tracked in the preceding modules to plan safe navigation effectively.

#### 2.1.6. Region Tracking Module

This module equips the system with the capability to detect and continuously monitor the position of specific regions or objects within the image feed. In autonomous driving, for example, such a module might be used to track the movement of vehicles or pedestrians, employing methods like Kalman filtering or optical flow algorithms to predict their future positions and plan a safe navigation path. Similarly, in the context of sea ice, tracking regions is essential for understanding the dynamics of ice floes and icebergs, which are critical for ensuring safe navigation in polar waters.

The module typically employs techniques such as object detection using convolutional neural networks (CNNs) combined with motion estimation methods like the Lucas–Kanade optical flow or the Particle Filter. These techniques allow the system to accurately follow the movement of ice floes or icebergs across successive frames in the image feed. The input for this module consists of segmented individual ice floes or objects, which have been identified in earlier stages of processing. The output is the tracked velocity and trajectory of these ice floes or icebergs, providing information for predicting their future positions and ensuring that the vessel can navigate safely around them. This continuous monitoring is important for preventing collisions and managing the dynamic environment of polar waters.

#### 2.1.7. Ice Load Prediction Module

Ice load prediction, traditionally carried out using GPU-based event mechanics (GEM) models [45] is a critical process that forecasts the pressures and forces exerted by ice on a vessel. These vary significantly depending on environmental factors such as ice thickness, density, and floe size. This module employs advanced AI models that integrate both historical (time-averaged) data and real-time (time-dependent) data to simulate and predict the dynamic and static forces acting on ships [45]. The AI algorithms utilize machine learning techniques, such as regression analysis and neural networks, to model the complex interactions between a vessel and the surrounding ice. These models consider various parameters, including ice velocity, temperature fluctuations, and the mechanical properties of the ice, to generate accurate predictions.

The input for the ice load prediction module is the data on tracked ice floes, which are derived from the object tracking modules. These data include detailed characteristics of the ice floes, such as size, shape, and movement patterns. The module then processes this information to output precise predictions of ice loads based on the floe characteristics. These predictions are used to support navigational decisions and ensure the safety of vessels operating in ice-covered waters. By leveraging AI-driven predictive analytics, this module enhances the vessel’s ability to anticipate and mitigate the risks associated with ice interactions.

## 3. Module Implementation

The deployment of modules of this architecture has become increasingly accessible due to the availability of open-source, user-friendly AI tools such as Roboflow [48] and Google Colab [49], enabling engineers with minimal coding experience to participate in the design and development process. This work demonstrates how these AI tools can be leveraged to prototype and implement the initial module of the architecture, showcasing a streamlined approach to integrating advanced technologies into practical applications.

### 3.1. Model Selection

The AI models used in image classification need to be trained using labeled datasets. The size of the dataset directly affects the accuracy of the trained AI model. The higher the number of images, the better the training accuracy of the network. Widely used AI models such as YOLOv8 [25], PSPNet [13], ICENET [11], and DeepLabv3+ [46] are typically trained using datasets with millions of images, such as ImageNet [50].

In this study, the **“YOLOv8n-cls”** model with 2.7 M parameters was selected due to its ability to perform fast inferences while operating in resource-constrained environments, such as being an on-board system on a ship [51,52]. The smaller model is specifically chosen to ensure that inference times remain low, allowing for near real-time decision making during navigation without overloading the available computational resources. Larger models, though more accurate, would require significant processing power and could slow down the system’s response time. Therefore, the trade-off between speed and resource efficiency makes the smaller YOLOv8n-cls model more suitable for this application.

### 3.2. Dataset Preparation

The training dataset should have at least 10,000 images to reach the required accuracy. Therefore, a labeled dataset of 5000 images was created from different dataset sources. Half of the images were taken from public sources such as YouTube videos and public images that can be downloaded from Google. The other half of the images were from the Americam Bureau of Shipping (ABS) and the Canadian Coast Guard (CCG) data repositories. After the images were collected, they were resized to a common size and augmented to increase the size of the dataset to reach 10,000 images. This 10,000-image dataset was used to train the image classification modules in the proposed system architecture. The dataset sizes vary due to ABS data, and the training split percentages remain consistent across experiments, maintaining uniformity as shown in Table 1.

### 3.3. Image Classification Module-I (ICM-I)

The ICM-I requires a fast processing AI-based algorithm that can handle a real-time image feed coming from on-board cameras. Therefore, once trained, the AI module should be able to infer the correct class in the range of milliseconds. A pre-trained **“YOLOv8n-cls”** multi-label classification model was used to implement the new model.

### 3.4. Labeling

Labeling involves categorizing images into specific classes to prepare them for use in the **“YOLOv8n-cls”** classifier. This process begins by organizing the images into five distinct classes, each represented by a separate folder named according to its class. The entire dataset is first divided into three main folders as follows: training, testing, and validation. To do this, 80% of the images were allocated to the training folder, 10% to the validation folder, and the remaining 10% to the testing folder. Within each of these main folders, five subfolders were created, corresponding to the following classes: forward_looking, side_looking, stern_looking, lighting_condition, and irrelevant. The Roboflow image annotation tool was used to initially classify the dataset into five classes for ICM-I.

### 3.5. Image Prepossessing

**Figure 5:**
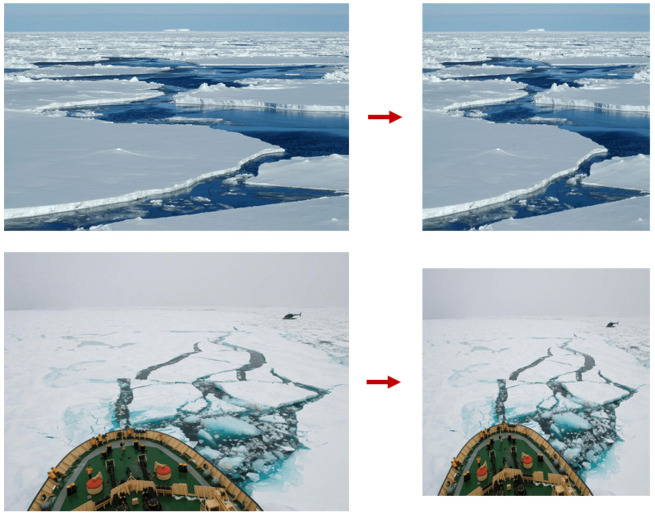
Image preprocessing carried out on ICM-I. The two rows of forward_looking images (original on the **left**) were resized (on the **right**) to 640 × 640 pixels.

Image preprocessing is the process of preparing images for input into a neural network by ensuring they are in a common format and a fixed size. This standardization is crucial as it facilitates efficient and consistent neural network training by providing uniform input data. In this study, the entire dataset of 5000 images was resized to 640 × 640 pixels. This resizing step ensures that all images have the same dimensions, allowing the neural network to process them more effectively and reducing the computational complexity during training, see Figure 5.

### 3.6. Image Augmentation

In image classification tasks, especially when dealing with smaller, custom datasets, data augmentation is a commonly employed strategy to enhance model performance. Image augmentation is a technique used to artificially expand the size of a dataset by applying various transformations to existing images. This process generates new, diverse training samples, which helps improve the robustness and generalization capabilities of the model. Common image augmentation methods include simple geometric transformations like rotation and scaling, horizontal flipping, color space adjustment, and random cropping. These techniques introduce variability in the dataset, allowing the model to learn from a broader range of scenarios and reducing the risk of overfitting.

**Figure 6:**
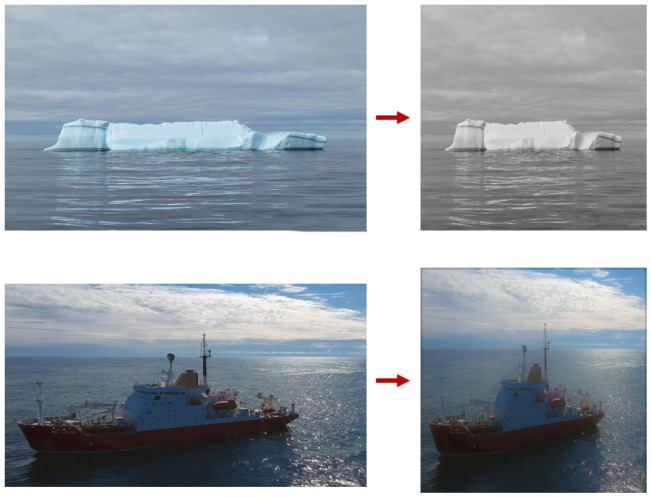
Image augmentation carried out on preprocessed images from the dataset. The **top** row indicates the grayscale augmentation. The **bottom** row indicates the addition of noise to the image. The images were used with consent from Envi.

In this study, for ICM-I, custom image augmentation was performed using a Jupyter Notebook script to expand the dataset from 5000 to 10,000 images. The script generated three additional variants for each original training example, effectively increasing the diversity of the dataset. The augmentation process included applying grayscale transformation to 15% of the images to simulate varying lighting conditions, introducing blur with a maximum radius of 2.5 pixels to mimic camera focus variations, and adding noise up to 0.1% of the pixels to replicate sensor noise. An example is shown in Figure 6. These augmentations were designed to enhance the model’s robustness and generalization by exposing it to a broader range of visual conditions.

### 3.7. Training

Training the ICM-I model was conducted using the following three platforms: the Roboflow tool [48], Google Colab [49], and the Compute Canada computing cluster [53]. The dataset used for ICM-I consists of five classes. For optimal model performance, it is important to ensure class balancing, meaning each class should have an equal number of images before training begins. Additionally, all images should be resized to a uniform size to improve training accuracy. In some cases, increasing the dataset size may be necessary to achieve better accuracy.

Roboflow, a graphical tool, offers multiple built-in functions for dataset preprocessing, augmentation, and health checks, including class balancing. This tool enables the rapid preparation of a sample dataset for training an image classifier. If any inaccuracies arise, they can be easily identified and corrected within Roboflow. Once the essential characteristics of the dataset are established, the training process can be scaled up using larger datasets on platforms like Google Colab and Compute Canada. This approach streamlines the entire data preparation and training process, reducing the need for rework and improving the overall efficiency of model development.

#### 3.7.1. Roboflow Training

Roboflow is a graphical tool which was created to ease computer vision tasks in the field of deep learning. The graphical self-guiding nature of the tool enables engineers with minimal coding experience to develop deep learning models for their specific needs. Comprehensive documentation and tutorials are also available for neural network model training [54].

In Roboflow training, the image dataset was uploaded to the Roboflow multi-label classification function. A dataset of 1000 public images was uploaded to Roboflow, preprocessed as Section 3.5, and augmented as Section 3.6 to increase the number of images to 2400. The training was carried out using 2100 training images, 200 validation images, and 100 testing images. Only public images were used since all the image data uploaded to Roboflow goes into the public Roboflow universe database. The neural network model used in this classification was *Roboflow 2.0 Multi-label Classification.*

In Roboflow training, the image size has been set to 640 × 640. The number of epochs, and the dataset split into training, testing, and validation have been automatically determined by Roboflow to maximize model accuracy.

#### 3.7.2. Google Colab Training

The model training process was conducted using Google Colab, where the entire workflow was executed within a Python script housed in a Colab notebook. A publicly available dataset, previously curated and hosted on Roboflow, was imported directly into the Colab environment. Utilizing this dataset, the YOLOv8 model was trained within the Colab notebook, enabling a direct comparison of its accuracy against the *Roboflow 2.0 Multi-label Classification* model.

To fine-tune the model and achieve results comparable to those obtained with Roboflow, various parameters, such as the number of training epochs and image sizes, were adjusted iteratively. A key difference from the Roboflow training environment is that, when training the YOLOv8 model in Colab, users must explicitly define parameters such as image size and the number of epochs. These parameters significantly influence the model’s performance and are crucial for tailoring the model to specific tasks.

The Colab notebook used for this training process is publicly accessible and can be found in this link—https://colab.research.google.com/drive/1BMObXc5-jvQwFxfVz_MJSC_0sSuZ1q3c?usp=sharing (accessed on 13 May 2024). However, it is important to note that due to the resource limitations inherent in Google Colab, where individual allocations are constrained, the training process could not be fully completed as initially planned. Although the script was configured to run for 1000 epochs, the session was automatically terminated after approximately 400–500 epochs, preventing the completion of the full training cycle. Despite this limitation, the model’s performance at the interrupted state still provided valuable insights for the study.

#### 3.7.3. Compute Canada Training

In the compute Canada Jupiter notebook, unlike Roboflow, the resources allocated are not limited, and the whole set of epochs can be run from Compute Canada. In Compute Canada, the model needs to be run with its terminal by executing the following commands to set up the environment. The dataset must be uploaded to Compute Canada using the Globus file transfer tool to train the model. After that, the YOLOv8 training command should be run in the same terminal to initiate model training.

### 3.8. Testing

Model testing is a critical phase in evaluating the performance and generalization capability of a trained AI model. For the YOLOv8 model, testing was conducted using a set of images that were not part of the training data, ensuring that the model’s ability to handle unseen data was effectively assessed. The testing process involved several steps, beginning with the selection of a specific subset of images from the original dataset.

During the Roboflow training of the ICM-I model, 100 images were selected and reserved exclusively for testing purposes. Similarly, the YOLOv8 model, trained in the Google Colab environment, was also evaluated using 100 testing images. These images were carefully chosen to represent a diverse range of scenarios and conditions that the model might encounter in real-world applications.

For a more comprehensive evaluation, the complete model training conducted on Compute Canada utilized an expanded testing set comprising 300 images. This larger testing set provided a more robust assessment of the model’s performance across a broader spectrum of conditions, allowing for a deeper analysis of the model’s strengths and potential areas for improvement.In summary, the testing phase ensures that the model is rigorously evaluated using a variety of images that it has not previously encountered, with different testing sets employed at various stages of the development process to validate the model’s accuracy and reliability.

### 3.9. Image Classification Module-II (ICM-II)

Following the initial processing in ICM-I, the forward-looking images are directed to ICM-II for further categorization. In this module, the images are classified into three specific categories as follows: ice_images, open_water, and objects. This classification step is essential for refining the analysis of the forward-looking visual data and ensuring the accurate detection of relevant environmental conditions. The classification in ICM-II is performed using the pre-trained YOLOv8 model, which was introduced in Section 3.1. The output from ICM-II is critical for subsequent modules that handle specific tasks such as semantic segmentation, instance segmentation, and object tracking.

### 3.10. Labelling

Similar to the process used in ICM-I, the initial image database of 3000 images was augmented to a total of 6000 images to enhance model training. These images were meticulously labeled using the Roboflow image annotation tool for the Roboflow training sessions. For the training conducted on Compute Canada, the images were manually organized and assigned to specific training, testing, and validation folders, ensuring a structured and effective dataset preparation for the model training process.

### 3.11. Image Pre-Processing and Augmentation

Image preprocessing and Augmentation are carried out similarly to ICM-I.

### 3.12. Training

The model training process for ICM-II was conducted following the same procedure as used for ICM-I. However, a key distinction in ICM-II is that the model was trained to classify images into three specific classes—ice_images, open_water, and objects—as opposed to the five classes used in ICM-I. This adjustment reflects the different classification requirements for ICM-II, focusing on a more targeted set of categories used to optimize the system’s performance in these particular areas.

## 4. Results

Comparative results from the ICM-I and ICM-II, generated from Roboflow, Google Colab, and Compute Canada, are presented in this section.

### 4.1. Roboflow Results

**Figure 7:**
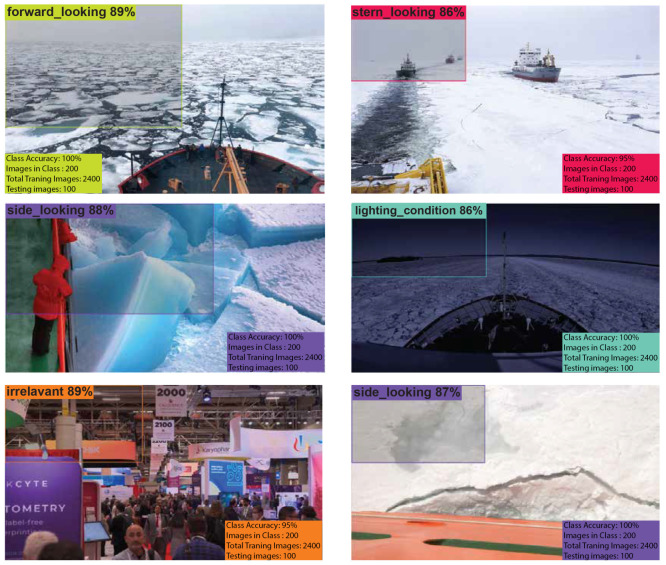
ICM-I results using Roboflow, with percentages indicating the model’s confidence in predicting the correct class for each image.

**Figure 8:**
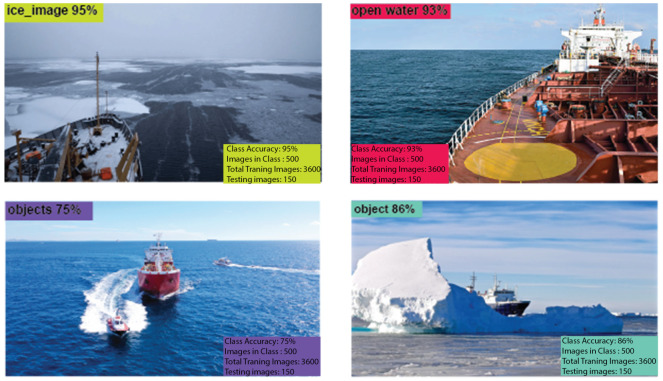
ICM-II results using Roboflow, with percentages indicating the model’s confidence in predicting the correct class for each image.

The public dataset used 1000 images and augmented 2400 images, resulting in a 98.8% validation accuracy as shown in Table 2. The visualization tool in Roboflow can be used to predict the classes of the images. Example of Roboflow results for ICM-I is shown in Figure 7 and ICM-II results are shown in Figure 8.

### 4.2. Google Colab Results

The Roboflow-prepared dataset was directly imported into Google Colab for training the YOLOv8 model. The model was configured to run for a maximum of 1000 epochs; however, due to resource limitations in the Colab environment, the training process was automatically halted after 514 epochs. Despite this, the model reached a point of optimal performance at epoch 414, where the best-performing model was saved.

Throughout the training process, the YOLOv8 model utilized approximately 0.377 GB of GPU memory and processed batches of 132 images at a speed of 7.07 iterations per second. The output from this training indicated a top-1 class accuracy of 96% and a top-5 class accuracy of 100% across all classes.

- **Top-1 class accuracy** refers to the percentage of instances where the model’s highest-confidence prediction (its first guess) matched the correct class label.
- **Top-5 class accuracy** measures the percentage of instances where the correct label was among the top five predictions made by the model.

These high accuracy values reflect the model’s strong capability to correctly classify images, often identifying the correct class immediately with very high confidence.

For validation, the model was tested using a set of 100 images that were withheld from the training process. The validation results were equally strong, with a top-1 class accuracy of 98% and a top-5 class accuracy of 100%. These results confirm the model’s robustness and its strong ability to generalize in response to new, unseen data.

In summary, the YOLOv8 model trained on the Roboflow-prepared dataset demonstrated high levels of accuracy and efficiency, even within the constraints of the Google Colab environment. The results underscore the model’s suitability for precise image classification tasks, with its performance remaining strong despite the early termination of the training process.

#### 4.2.1. Confusion Matrix

**Figure 9:**
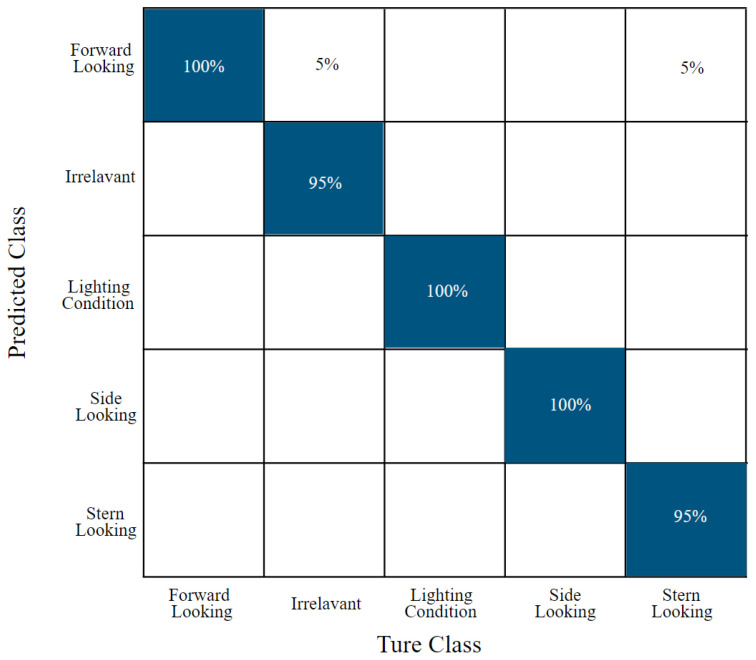
The confusion matrix generated from YOLOv8 model training in Colab.

The confusion matrix in Figure 9 from the Colab training session reveals that the YOLOv8 model performs strongly across most categories. Notably, the model achieved 100% accuracy in identifying **Lighting Condition** and **Side-Looking** images, as well as 100% accuracy in classifying **Forward-Looking** images correctly. Additionally, 95% of images labeled as **Irrelevant** were classified correctly, and 95% of **Stern-Looking** images were accurately predicted. These results indicate the model’s effectiveness in recognizing distinct image categories, particularly those with clear features, such as lighting variations or distinct camera perspectives. The perfect accuracy in some categories highlights the robustness of the model’s feature extraction and classification abilities in these cases.

However, some misclassifications were observed, indicating areas where the model could be improved. Specifically, 5% of **Forward-Looking** images were misclassified as **Irrelevant**, while another 5% were incorrectly categorized as **Stern-Looking**. These errors suggest that the model occasionally struggles to differentiate between similar perspectives or ambiguous image features. Such misclassifications underscore the need for refining the model’s ability to distinguish between closely related classes, perhaps through enhanced feature representation or additional training data tailored to these challenging categories. Overall, the confusion matrix highlights the model’s classification performance while providing insights into specific areas for refinement to further enhance its accuracy and reliability in real-world applications.

#### 4.2.2. Model Training Accuracy Graph

**Figure 10:**
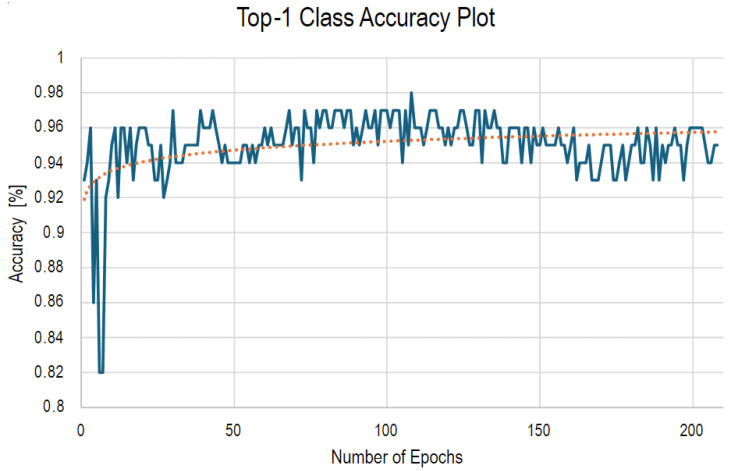
The class training accuracy plot stopped at 200 epochs in Google Colab due to resource constraints.

The graph presented in Figure 10 illustrates the top-1 class accuracy over the course of training epochs for the YOLOv8 model in Google Colab. This plot provides several key insights into the model’s performance during the training process.

1. Initial Accuracy Improvement
  - At the start of training, there is a noticeable rapid improvement in accuracy, which is typical as the model begins to learn and adjust its parameters. The accuracy starts at a lower point but quickly rises to around 92–94% within the first few epochs.
2. Fluctuations During Training
  - After the initial improvement, the accuracy graph shows some fluctuations. These fluctuations indicate that while the model continues to learn, it is also adjusting and re-adjusting its parameters, which can cause temporary dips in accuracy. This is a normal part of the training process, especially when dealing with complex datasets.
3. Stabilization of Accuracy
  - As training progresses, the accuracy stabilizes around 94–96%. This suggests that the model has found a relatively optimal set of parameters and is consistently performing well across the training dataset. However, the slight fluctuations indicate that the model is still exploring the parameter space.
4. Trend and Long-Term Behavior
  - The orange dotted line represents a trend line, showing the general direction of the accuracy over time. The trend indicates a slight improvement over time, even though the model experiences some volatility in accuracy from epoch to epoch.
5. Early Stopping in Colab
  - It’s important to note that this training was conducted in Google Colab, where resource constraints resulted in the training being stopped prematurely. The graph only shows the first 200 epochs, but the training was intended to run for up to 1000 epochs. This early stopping means that while the model was showing signs of continued learning, it was not able to fully converge to its final accuracy potential within the Colab environment.

The accuracy graph provides valuable insights into the training process of the YOLOv8 model. The initial rapid improvement, followed by fluctuations and eventual stabilization, indicates that the model is learning effectively but was cut short due to resource limitations in Google Colab. The trend suggests that further training could have led to even better performance, but within the given constraints, the model still achieved a strong level of accuracy. This graph highlights the importance of adequate computational resources in fully training deep learning models to their maximum potential.

### 4.3. Compute Canada Training Results

Since there were resource limitations in Colab when carrying out model training, the process was switched to Compute Canada. The dataset was significantly augmented to include 10,000 images for ICM-I and 6000 images for ICM-II. The model was then trained for 1000 epochs using these expanded datasets. The results were summerized in Table 3.

#### 4.3.1. Confusion Matrix

**Figure 11:**
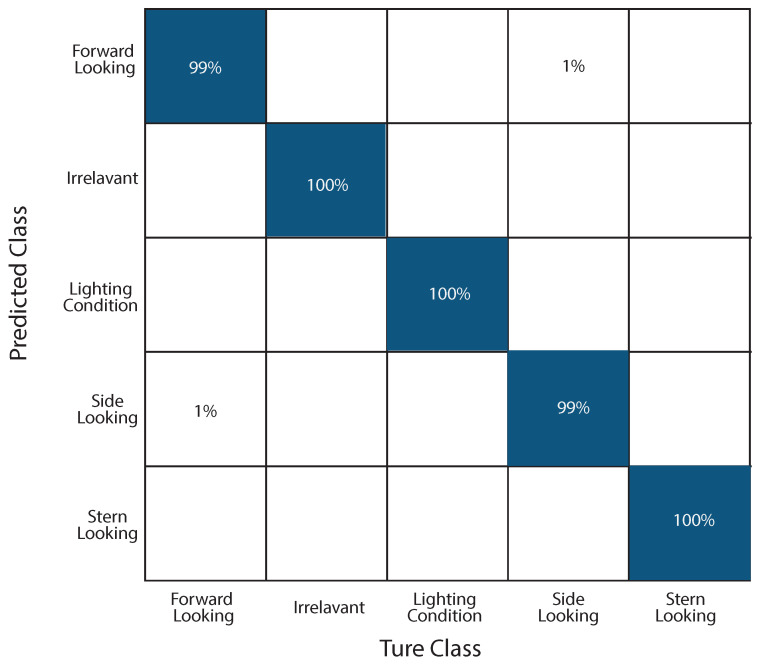
Confusion matrix for the 10,000-image dataset with 1000 training epochs for ICM-I.

The confusion matrix from the Compute Canada training session shown in Figure 11, utilizing a larger dataset and extended training period, demonstrates significant improvements in the model’s performance. The results show near-perfect accuracy, with 99% for **Forward-Looking** and **Side-Looking** classes, and 100% for **Irrelevant**, **Lighting Condition**, and **Stern-Looking** classes. This indicates the model’s ability to effectively distinguish between categories with minimal errors. Compared to the Colab training session, which showed minor misclassifications (e.g., **Forward-Looking** images occasionally classified as **Irrelevant** or **Stern-Looking**), the Compute Canada results exhibit more consistent and accurate classifications.

These improvements underscore the importance of a larger training dataset and an extended training duration. The increased data volume and additional training epochs allowed the model to generalize better across diverse scenarios, significantly reducing confusion between similar classes. The Compute Canada results highlight the critical role of computational resources and data availability in enhancing the performance of deep learning models for complex classification tasks.

#### 4.3.2. Model Training Accuracy Graph—ICM-I

**Figure 12:**
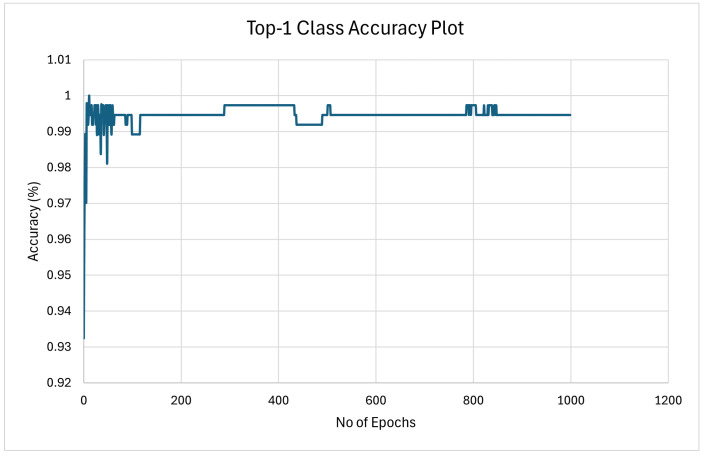
Model training accuracy graph for the 10,000-image dataset with 1000 training epochs.

The top-1 class accuracy plot from the Compute Canada training is shown in Figure 12, which involved a more extensive dataset and a longer training duration. It shows significant improvements in both stability and overall accuracy compared to the Colab training results. The Compute Canada plot demonstrates an accuracy that consistently hovers around 99–100% throughout the training process, indicating that the model has effectively learned to classify images with minimal error.

In contrast, the Colab training plot showed more fluctuations and a final accuracy stabilization around 94–96%, reflecting the limitations imposed by the smaller dataset and fewer training epochs due to resource constraints. The longer training time and increased data in Compute Canada allowed the model to fine-tune its parameters more effectively, resulting in fewer misclassifications and a more reliable performance. This comparison highlights the importance of sufficient computational resources and comprehensive datasets in achieving optimal model accuracy, as demonstrated by the improved performance in the Compute Canada environment. The fast stabilization and subsequent fluctuations in accuracy suggest that the learning rate or weight adjustment schedule could benefit from further optimization. Future work could explore slower weight changes, such as using a learning rate decay schedule or gradient clipping, to achieve smoother learning and mitigate accuracy drops.

#### 4.3.3. Confusion Matrix—ICM-II

**Figure 13:**
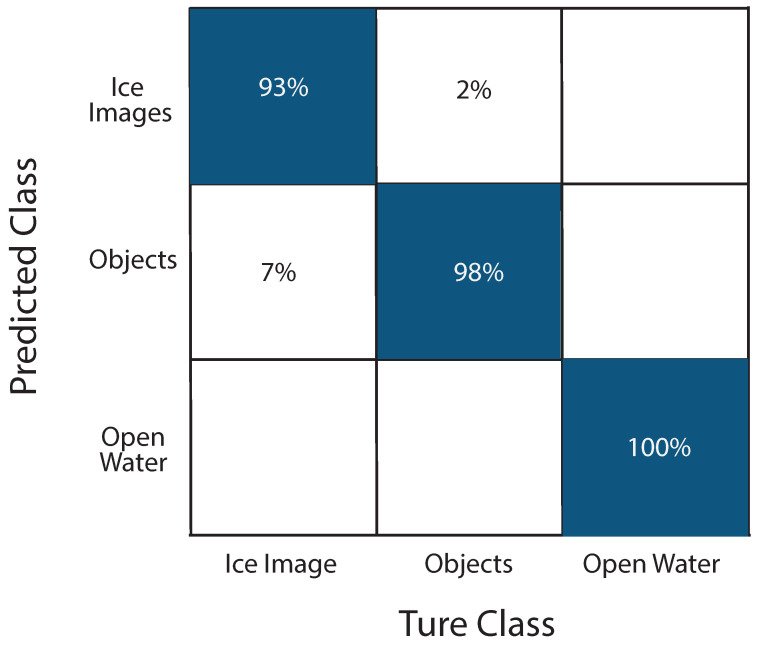
Confusion matrix for the 6000-image dataset with 1000 training epochs for ICM-II.

The confusion matrix generated for Image Classification Module II (ICM-II) as shown in Figure 13, trained using Compute Canada’s resources, provides a clear insight into the model’s performance in categorizing images into three classes, **Ice Images**, **Objects**, and **Open Water**. The key insights from this are as follows:

1. High Accuracy in **Ice Images** and **Objects**:
  - The model correctly classified 93% of the images that truly belong to the **Ice Images** class, with a 7% misclassification rate where some **Ice Images** were incorrectly identified as **Objects**.
  - For the **Objects** class, the model achieved a high accuracy of 98%, with a small portion (2%) of **Objects** being misclassified as **Ice Images**
2. Accurate Classification of **Open Water** Images:
  - The **Open Water** class shows accurate classification, with 100% of the images correctly identified as **Open Water** by the model. This indicates that the model is highly reliable in distinguishing **Open Water** images from the other categories.
3. Areas for Improvement
  - The confusion matrix reveals that the most significant confusion occurred between **Ice Images** and **Objects**. The 7% of **Ice Images** misclassified as **Objects** and the 2% of **Objects** misclassified as **Ice Images** suggest that the model might benefit from further refinement, particularly in distinguishing between these two classes. This could involve more extensive training data or additional feature extraction techniques to help the model better differentiate between ice structures and other objects.

Overall, the ICM-II model trained on Compute Canada demonstrates strong classification performance, especially in identifying **Open Water** images with 100% accuracy. The high accuracy rates for **Ice Images** and **Objects** are also commendable, although the confusion between these two classes highlights an area for potential enhancement in future training iterations. This confusion matrix provides a valuable assessment of the model’s current strengths and areas for refinement.

#### 4.3.4. Model Training Accuracy Graph—ICM-II

**Figure 14:**
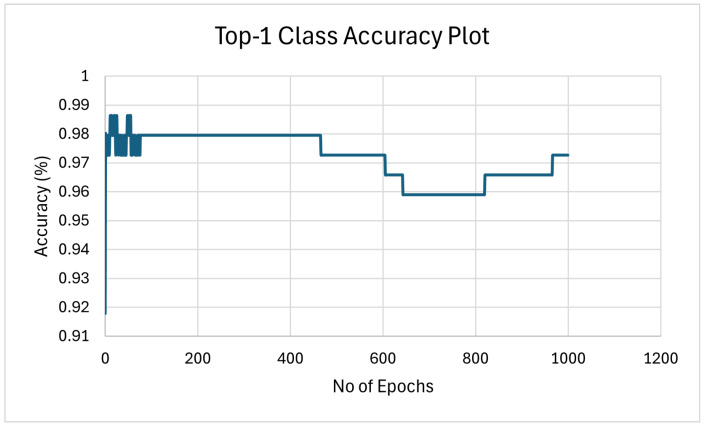
Model training accuracy graph for ICM-II for 1000 training epochs.

The top-1 class accuracy plot for the training of Image Classification Module II (ICM-II) in Figure 14 reveals key insights into the model’s learning process over 1000 epochs. Initially, the model’s accuracy stabilizes around 98%, showing that it quickly learns to classify the data effectively. However, as the training progresses, a noticeable drop in accuracy occurs, with the accuracy decreasing in steps after approximately 400 epochs.

This drop suggests that the model may have encountered challenges in further refining its classification ability, potentially due to overfitting or changes in the learning rate. The accuracy remains relatively consistent at around 97% for a large portion of the training but experiences further slight decmidrules before showing a gradual recovery towards the final epochs.

The final accuracy stabilizes slightly below 98%, indicating that while the model was able to recover some of its initial accuracy, it did not fully regain the peak performance observed at the beginning. This behavior suggests that although the model is generally robust, there may be room for improvement in terms of optimizing the training process, perhaps by adjusting the learning rate schedule, increasing the dataset size, or applying techniques to prevent overfitting. The observed decmidrule in accuracy after 400 epochs might suggest potential overfitting; however, further investigation is needed to confirm this and identify its root causes. Future work will examine factors such as learning rate schedules, dataset properties, and model adjustments to better understand and address this behavior, enabling a more robust training process.

## 5. Inference Speed Comparison Results

The objective of the ICM-I and ICM-II is to classify the image feed coming from the on-board camera in real-time. This classification allows the subsequent modules to operate efficiently, minimizing the processing delay caused by images that are difficult to process. The inference speed testing was carried out using Google Colab and a local laptop computer.

### 5.1. Google Colab

**Figure 15:**
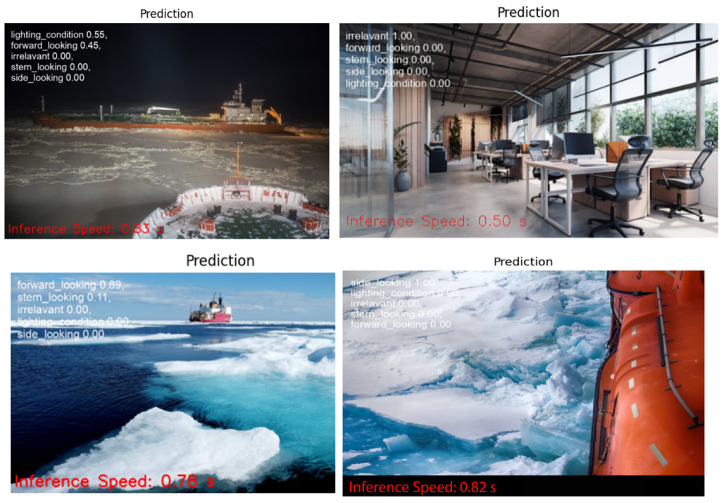
Inference speed results from ICM-I on Google Colab.

**Figure 16:**
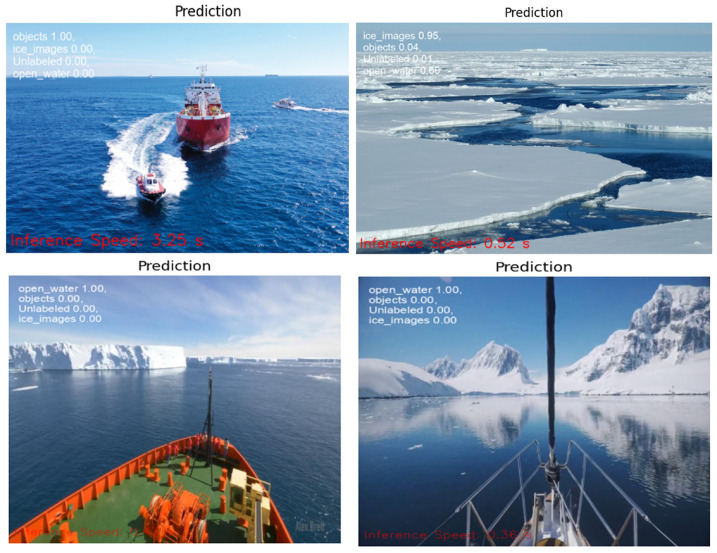
Inference speed results from ICM-II on Google Colab.

The trained model file from the Compute Canada platform is downloaded and uploaded into a Google Colab notebook. The notebook uses the YOLOv8 model function to obtain the inference on selected test images. The inference speed was calculated using the Python time function. Figure 15 and Figure 16 illustrates the results from Google Colab, where the inference speed is indicated at the bottom of each image. In Google Colab, the inference speed is notably fast, attributable to the high-performance GPUs available in the Colab environment.

### 5.2. Stand-Alone Laptop Computer

**Figure 17:**
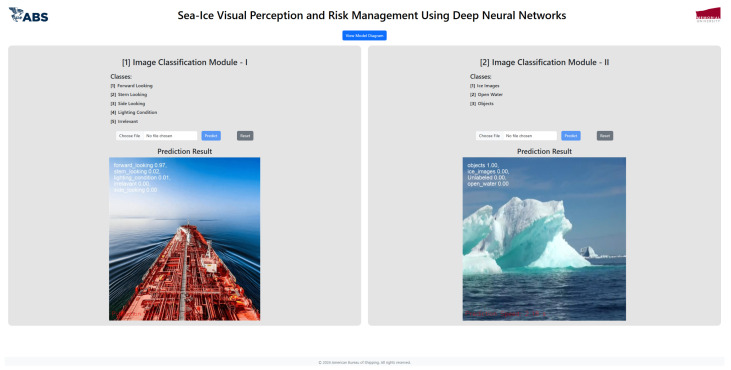
Model-testing web interface. The left side shows the ICM-I model test and the right side shows the ICM-II model test interface.

Given the onboard ship environment, it is crucial to evaluate the system’s inference performance using a standard laptop. The laptop used for testing was equipped with an Intel i7 quad-core processor and 16 GB of RAM. A web-based application was developed using JavaScript and Python to facilitate model testing, as shown in Figure 17.

The inference speeds recorded were approximately five seconds per image, primarily due to the use of a CPU rather than a more powerful GPU, as seen in environments like Google Colab. Despite this, the YOLOv8 model achieved inference times within the range of seconds, which remains acceptable for real-time maritime applications. These results demonstrate that even when operating on a CPU, the system can still provide timely responses, making it viable for onboard deployment where computational resources may be limited. A summary of the inference results are shown in Table 4.

## 6. Complete Ice Navigation Architecture

The complete ice navigation architecture integrates multiple camera feeds from onboard cameras to provide real-time ice condition awareness and risk quantification. This architecture is designed to ensure that ships navigating icy waters can effectively monitor their surroundings, aiding ship captains and ice captains in making informed decisions to enhance safety.

The architecture processes the following five distinct image streams outputted from Image Classification Module I (ICM-I): forward_looking, side_looking, stern_looking, lighting_condition, and irrelevant. The forward_looking stream is further analyzed in Image Classification Module II (ICM-II) and then passed through semantic segmentation and instance segmentation modules for risk quantification and ice floe tracking. This process is crucial for identifying potential hazards directly in the ship’s path and assessing the associated risks.

The side_looking and stern_looking image streams are particularly important for calculating ice pressure and ice loads on the ship’s hull. Ice pressure refers to the forces exerted by ice on the vessel, which can vary depending on factors such as ice thickness, concentration, and movement. By analyzing the side and stern views of the ship, the system can assess the ice conditions around the vessel, providing valuable data on the ice forces that the ship may encounter.

Using state-of-the-art techniques in image processing and AI, the architecture can estimate ice loads by analyzing the size, density, and distribution of ice floes captured in these side and stern views. This information is critical for understanding the potential impact of ice on the ship’s hull and making real-time adjustments to the vessel’s course or speed to mitigate risks. The integration of these calculations into the overall navigation system enhances the ship’s ability to navigate safely through ice-covered waters, reducing the likelihood of damage due to ice impact.

## 7. Conclusions

This work proposed and partially implemented a deep learning architecture for ice risk management, which includes modules for ice classification, risk assessment, ice floe tracking, and ice load calculations. In this work, the focus was on the development and implementation of the image classification modules I and II. To train these modules, a dataset comprising 15,000 images was created, drawing from publicly available sources, as well as datasets provided by the American Bureau of Shipping (ABS) and the Canadian Coast Guard (CCG).

The YOLOv8 classification model was selected for this work due to its superior speed and efficiency compared to other state-of-the-art methods. The training process was carried out in three stages as follows: initially using Roboflow, followed by Google Colab, and finally on Compute Canada’s powerful computing resources. This multi-stage approach was incoporated to iteratively refine the dataset and improve model performance in a robust final training stage on the Compute Canada computing cluster.

The results demonstrated that Image Classification Module I achieved a validation accuracy of 99.4%, while Image Classification Module II reached 98.6% accuracy. The inference speed of the YOLOv8 model was also evaluated, with results showing that inference time on Google Colab was less than 1 s. Additionally, a stand-alone web application was developed to test inference speed further, and it was found that inference on an 11th-generation Intel i7 2.3 GHz processor took less than 3 s.

Several future directives have been identified to advance this work. First, the dataset of ice images should be expanded to enhance the accuracy of the model further. Completing the full architecture with the necessary modifications is also a priority to realize a comprehensive ice navigation and risk management system. The next critical steps involve implementing the semantic segmentation and instance segmentation modules. These modules will require the careful labeling of newly collected datasets to ensure precise and accurate training. Given the solid foundation established by this work, these future efforts are essential to develop a fully functional and effective system for ice navigation in challenging polar environments.

## Figures and Tables

**Table 1:** Overview of the datasets used for ICM-I and ICM-II, including the number of raw and augmented images, the dataset split percentages for training and validation, the validation accuracy of the trained models, and the respective training platforms.

Module	Dataset	Raw Images	Aug. Images	Train Split (%)	Accuracy	Platform
ICM-I	Public	1000	2400	80-10-10	98.0%	Roboflow
ICM-I	Public + ABS	5000	9180	88-8-4	99.5%	Compute Canada
ICM-II	Public	1500	3633	80-10-10	98.6%	Roboflow
ICM-II	Public + ABS	1750	4598	88-8-4	98.5%	Compute Canada

**Table 2:** The initial Roboflow results for ICM-I and ICM-II.

Module	Model	No. of Images	Validation Accuracy
ICM-I	Roboflow 2.0 Multi-label Classification	2400	98.8%
ICM-II	Roboflow 2.0 Multi-label Classification	3633	98.8%

**Table 3:** Compute Canada results for ICM-I and ICM-II.

Module	Model	No. of Images	Validation Accuracy
ICM-I	YoloV8n-cls model	9180	99.45%
ICM-II	YoloV8n-cls model	6633	99.8%

**Table 4:** Inference speed comparison results for ICM-I and ICM-II.

Module	Test Images	Inference Time (s)	
		Google Colab	Laptop
ICM-I	image-1 image-2 image-3 image-4	0.83 0.50 0.78 0.82	3.23 4.35 3.11 2.01
ICM-II	image-1 image-2 image-3 image-4	3.25 0.52 0.43 0.36	4.23 1.35 2.11 4.01

## Data Availability

Data are contained within the article.
